# Sociodemographic and occupational influences on health professionals’ quality of life

**DOI:** 10.1590/0034-7167-2024-0010

**Published:** 2024-11-22

**Authors:** Alberto de Oliveira Redü, Daiani Modernel Xavier, Marcela Amaral Daoud, Pâmela Kath de Oliveira Nörnberg, Natália Sperli Geraldes Marin dos Santos Sasaki, Geovane Mendes Farias, Luciano Garcia Lourenção

**Affiliations:** I Universidade Federal do Rio Grande. Rio Grande, Rio Grande do Sul, Brazil; II Faculdade de Medicina de São José do Rio Preto. São José do Rio Preto, São Paulo, Brazil

**Keywords:** Quality of Life, Health Personnel, Intensive Care Units, Working Conditions, Work., Calidad de Vida, Personal de Salud, Unidades de Terapia Intensive, Condiciones de Trabajo, Trabajo.

## Abstract

**Objective:**

To analyze the sociodemographic and occupational influences on health professionals’ quality of life.

**Method:**

This descriptive-exploratory, cross-sectional, analytical, and quantitative study addressed 94 health workers, including nursing technicians, nurses, and physical therapists working in Intensive Care Units in a town in the extreme south of Brazil in 2023. The student’s t-test and Spearman correlation were used.

**Results:**

A significant positive correlation was found between being a woman and the psychological domain and between income and the social and environmental domain while working hours were inversely related to general QoL. Additionally, workload negatively impacted the physical, psychological, and general QOL, furniture negatively influenced the psychological domain, and equipment was negatively associated with the physical and psychological domain.

**Conclusion:**

The characteristics of the work environment interfere with several areas of quality of life.

## RESUMEN

**Objetivo:**

Analizar las influencias sociodemográficas y ambientales de trabajo en la calidad de vida de profesionales de la salud.

**Método:**

Se trata de un estudio descriptivo exploratorio, transversal, analítico y cuantitativo. Estuvo constituido por 94 profesionales de la salud, siendo técnicos en enfermería, enfermeros y fisioterapeutas, actuantes en Unidades de Terapia Intensiva, en un municipio del extremo sur de Brasil, en el año de 2023. Fue utilizado el test *t* de Student y la Correlación de Spearman.

**Resultados:**

Se encontró correlación positiva significativa en la variable sociodemográfica del sexo femenino con el dominio psicológico; la renta se asoció positivamente con el dominio social y el ambiente; las horas de trabajo se relacionaron inversamente con la CV general; el volumen de servicio interfirió negativamente en el dominio físico, psicológico y CV general; el mobiliario influenció de manera negativa el dominio psicológico; y, los equipamientos se relacionaron negativamente con el dominio físico y psicológico.

**Conclusión:**

se verificó que las características del ambiente de trabajo interfieren en diversos dominios de la calidad de vida.

## INTRODUCTION

In Brazil, health professionals working in Intensive Care Units (ICUs) are constantly subjected to work overload due to the need to perform multiple tasks^(1)^. Work in such an environment is organized according to complex tasks performed with critical patients demanding specific and highly complex care. Hence, the care model in an ICU is customized according to each patient’s needs and demands^(2,3)^.

Due to recent economic conditions, health workers have struggled to meet work demands besides performing domestic chores while allocating little time to leisure^(4)^. Work overload arising from working conditions^(5)^, unhealthy environments, and a lack of equipment, materials, or poor infrastructure^(6)^, elements necessary to meet demands, is harmful to workers’ health, contributing to a decrease in their quality of life^(7)^.

Quality of Life (QoL) is conceptualized “as the individual’s perception of their position in life in the context of the culture and value systems in which they live and in relation to their objectives, expectations, standards and concerns”^(8)^. Note that the concept is comprehensive, permeating physical and psychological aspects, independence level, social relationships, and personal beliefs^(9)^. Although some studies show that the quality of life of health professionals generally presents good scores, it was highly affected by the COVID-19 pandemic, in which there were many stressors, causing the relationship between stress and quality of life to become significant. Consequently, quality of life was negatively affected by factors such as low resilient levels, which also impacted the quality of the care delivered^(10,11)^.

The literature presents evidence of occupational issues affecting one’s quality of life. Studies conducted in Africa, Saudi Arabia, Malaysia, and Vietnam, for instance, show that environmental factors in the workplace and outbreaks of diseases such as Ebola and COVID-19 affect workers’ quality of life. Situations that threaten an individual’s quality of life or the experience of poor quality of life make it considerably challenging to deal with a high demand of patients or inadequate infrastructure conditions, predisposing professionals to exhaustion, leading to Burnout Syndrome, stress, and low resilience to cope with everyday problems^(4,12-14)^.

Several studies indicate that the quality of life of health workers is related to burnout syndrome, stress, resilience, and strategies to deal with these conditions. However, such studies fail to show how the workplace’s social and environmental characteristics interfere with professionals’ daily lives outside the workplace. Thus, we assume that the work environment’s limitations interfere with how occupational tasks are performed, how care is provided to patients, and how workers manage to meet their jobs’ demands more promptly. Such factors impact the workers’ physical and mental health as they lead to feelings of impotence when workers cannot provide care they believe to be appropriate due to inadequate conditions.

Healthcare is one of the pillars of current society, and adequate conditions are required for health workers to satisfactorily meet the population’s needs. Additionally, an unfavorable work environment affects QoL in the most diverse spheres. Thus, given the previous discussion, the following guiding question is proposed: What are the sociodemographic and environmental aspects of the workplace affecting the quality of life of health professionals?

## OBJECTIVE

To analyze the sociodemographic and occupational influences on the quality of life of health professionals.

## METHODS

### Ethical Aspects

The Institutional Review Board at the Federal University of Rio Grande (CEP-FURG) approved this study with Certificate of Presentation of Ethical Appreciation (CAAE). Consent was requested from the hospitals where the study was conducted, and the representatives of the sectors responsible for research in these institutions signed an authorization form. Likewise, the participants consented to participate in the study by signing two copies of a free and informed consent form; one copy was kept by the participant and the other by the primary author.

### Study Design and sampling

This is a descriptive-exploratory, cross-sectional, and correlational study with a quantitative approach^(15)^. It was conducted in two hospitals in the extreme south of Brazil. One is a university hospital, whose adult ICU has six beds, and the other is a philanthropic hospital with three ICUs. The ICUs of the second hospital provide care to uninsured individuals and individuals with health insurance. The general ICU has 10 beds, the Post-Operative Unit also has 10 beds, and the cardiac surgery and cardiological care ICU has nine beds.

The study population comprised 160 health professionals. Inclusion criteria were being a nursing technician, a nurse, or a physical therapist working in an ICU. The exclusion criterion was being on vacation or sick leave during data collection. A total of 124 health professionals remained after the criteria were applied. Next, the sample was calculated using StatCalc Epi Info version 7.2, with a 95% confidence level and a margin of error of 5%, resulting in a sample of 94 professionals. The Strengthening the Reporting of Observational Studies in Epidemiology (STROBE) was used to guide this study.

Given the post-pandemic context in which this study was conducted and considering that this paper is an excerpt of the dissertation “*Dor cervical e qualidade de vida: repercussões em profissionais de saúde de unidade de terapia intensiva*” [Neck pain and quality of life: repercussions on health professionals in intensive care units], we decided to include nursing and physical therapy professionals as they provided direct and immediate assistance. Hence, workers from both professions experience similar routines, such as lifting and moving patients in bed and performing daily tasks in the unit.

### Data Collection

First, the participants received clarification about the study’s objectives through a message sent to each unit’s WhatsApp group and then were personally invited by the researcher. The primary author collected data from April to August 2023 using a self-administered multiple-choice questionnaire, whose application took approximately 30 to 45 minutes.

The questionnaire addressed sociodemographic variables (function, sex, age, family income, working hours, and years of professional experience) and variables concerning the workplace’s characteristics (workload, furniture, equipment, and physical structure), which originated from the *Centro de Referência em Saúde do Trabalhador de Bahia de Ilha Grande* [Reference Center in Occupational Health in Bahia de Ilha Grande]^(16)^. Furthermore, the Whoqol-Bref was used for the dependent variable, which contains 26 questions rated on a five-point Likert scale, allocated into the physical, psychological, environmental, social relationships, and general quality of life domains^(9)^.

A pilot test was performed with the professionals from one work shift in one of the units to check whether the questionnaires presented semantic and structural validity. As no reformulation was required, these participants were included in the sample.

### Data Analysis

Data were entered and tabulated in the Statistical Package for the Social Science (SPSS), version 28.0. Depending on the data distribution, the quantitative variables were described by mean and standard deviation or median and interquartile range.

The analysis of the Whoqol-bref adopted the criterion in which the items in each domain were summed up and then divided by the number of items in the respected domain. Note that the recoding of questions 3, 4, and 26 was considered in the calculation^(9)^.

The Kolmogorov-Smirnov test was used to verify the normality of data. Next, the variables were described by absolute and relative frequencies. The Student’s t-test was used to compare the means, and the Spearman Correlation test was performed to verify associations between the ordinal variables. A 5% (p<0.05) significance level was adopted.

## RESULTS

Ninety-four health workers participated in this study. Table 1 shows the sample’s characteristics: most (88.3%) were women, 34.1% were between 30 and 39, and 31.9% had a family income between 1 to 2 times the minimum wage. Regarding weekly working hours, 67% worked 36 to 40 hours a week, 38.3% had 1 to 5 years of professional experience, and 63.8% were nursing technicians.

**Table 1:** Characterization of the sample including ICU health workers, Rio Grande, Rio Grande do Sul, Brazil, 2023 (n=94)

Variables	n	%
Sex		
Male	11	11.7
Female	83	88.3
Age group		
20 to 29	25	26.6
30 to 39	32	34.1
40 to 50	31	33.0
Older than 50	6	6.4
Family income		
Between 1 and 2 times the MW	30	31.9
Between 2 and 3 times the MW	22	23.4
Between 3 and 5 times the MW	23	24.5
Between 5 e 20 times the MW	20	20.2
Weekly working hours		
20 to 30 hours	18	19.1
36 to 40 hours	57	67.0
More than 40 hours	13	13.8
Professional experience		
0 to 12 months	9	9.6
1 to 5 years	36	38.3
6 to 9 years	15	16.0
More than 10 years	34	36.2
Profession		
Nursing technician	60	63.8
Nurse	21	22.3
Physical Therapist	13	13.8

*Note: MW: Minimum Wage R$1,320.00/mo ≈ USD 270.49/mo (1 USD = R$4.88).*

Table 2 presents the variables concerning the professionals’ work environment, quantified by qualifiers. Therefore, 52.1% of the professionals considered that they experienced work overload, 48.9% rated the unit’s furniture as regular, and the equipment was rated between good and regular, with each option achieving 44.7%.

**Table 2:** Characteristics of the work environment of the ICU health workers, Rio Grande, Rio Grande do Sul, Brazil, 2023 (n=94)

Variables	n	%
How do you evaluate your workload?		
Mild	1	1.1
Moderate	35	37.2
There is overload	49	52.1
Exhaustive	9	9.6
How do you evaluate the furniture in your unit?		
Excellent	2	2.1
Good	34	36.2
Regular	46	48.9
Poor	11	11.7
Very poor	1	1.1
How do you evaluate the equipment in your unit?		
Excellent	4	4.3
Good	42	44.7
Regular	42	44.7
Poor	6	6.4

Table 3 shows that the means ranged from 58.9 to 68.1 in the analysis of the Whoqol-Bref domains. The environment domain obtained the lowest score (58.9). It is composed of the following facets: Physical security and protection; Home environment; Financial resources; Health and social care; Availability and quality; Opportunities to acquire new information and skills; Participation in/and recreation/leisure opportunities; Physical environment (pollution/noise/traffic/climate), and transport. The social domain obtained the highest score (68.1), comprising the Personal relationships, Social support, and sexual activity facets.

**Table 3:** Mean scores obtained in the Whoqol-Bref domains, Rio Grande, Rio Grande do Sul, Brazil, 2023 (n=94)

Domains	Mean ± SD	Minimum/ Maximum
Physical	64.6 ± 13.5	35.7 - 100
Psychological	65.7 ± 15.1	25.0 - 95.8
Social Relations	68.1 ± 18.5	25.0 - 100
Environment	58.9 ± 13.5	28.1 - 90.6
General	60.1 ± 19.3	12.5 - 100

*SP: Standard Deviation*

**Figure 1:**
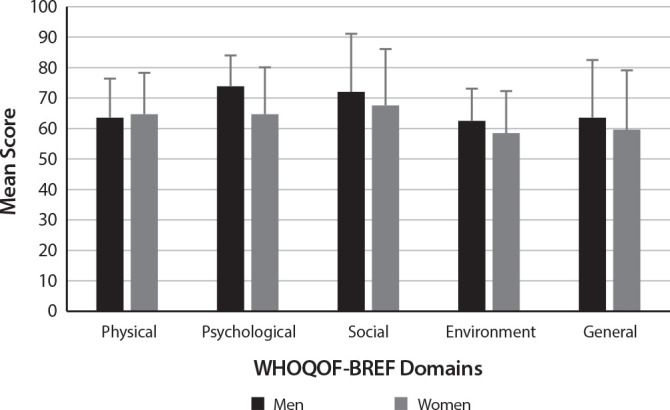
Assessment of the quality of life scores according to the participants’ gender, Rio Grande, Rio Grande do Sul, Brazil, 2023 (n = 94)

The analysis comparing the quality of life scores according to gender showed a significant difference only in the psychological domain (p=0.017), in which women obtained lower scores. Figure 1 shows that the remaining differences between the sexes (p>0.30) were not statistically significant.

Table 4 presents the results concerning the associations between sociodemographic and occupational variables with the Whoqol-bref domains. A statistically significant direct association was found between income and the social and environmental domains of the Whoqol-Bref, i.e., the higher the income, the higher the scores obtained in these domains. Working hours were inversely associated with the general quality of life, i.e., the longer the working hours, the lower the general Whoqol-Bref scores.

**Table 4:** Associations between quality of life scores and the sociodemographic and occupational variables, Rio Grande, Rio Grande do Sul, Brazil, 2023 (n=94)

Variable	WHOQOL-BREF Domains				
	Physical	Psychological	Social	Environment	General
Age	-0.09	0.14	-0.07	-0.09	-0.06
	(p=0.372)	(p=0.171)	(p=0.516)	(p=0.380)	(p=0.554)
Family Income	0.08	0.19	0.26	0.20	0.11
	(p=0.437)	(p=0.060)	(p=0.011)	(p=0.049)	(p=0.282)
Working hours	-0.05	-0.17	-0.12	-0.10	-0.27
	(p=0.655)	(p=0.112)	(p=0.253)	(p=0.349)	(p=0.009)
Working experience	-0.06	0.09	0.04	-0.01	-0.02
	(p=0.559)	(p=0.400)	(p=0.696)	(p=0.900)	(p=0.873)
Workload^*^	-0.25	-0.20	-0.18	-0.17	-0.31
	(p=0.015)	(p=0.049)	(p=0.081)	(p=0.093)	(p=0.002)
Furniture in your unit^*^	-0.18	-0.26	-0.18	-0.09	-0.10
	(p=0.092)	(p=0.012)	(p=0.075)	(p=0.399)	(p=0.337)
Equipment in your unit^*^	-0.22	-0.28	-0.12	-0.14	-0.17
	(p=0.030)	(p=0.006)	(p=0.251)	(p=0.191)	(p=0.110)
The physical structure of your unit^*^	0.04	0.09	-0.03	-0.04	-0.01
	(p=0.677)	(p=0.399)	(p=0.742)	(p=0.735)	(p=0.916)

Note: Spearman’s correlation coefficients were included in the table;

* the higher the score, the worse the assessment

An inverse significant association was found between workload and the physical, psychological, and general quality of life domains, indicating that the greater the workload, the lower the quality of life scores. Furthermore, a significant inverse association was found between the assessment of furniture in the unit and the psychological domain, showing that the worse furniture was considered (higher scores), the lower the scores obtained in this domain of quality of life (lower scores). Finally, a significant inverse association was found between the assessment of equipment in the unit and the physical and psychological domains, indicating that the worse equipment was considered, the lower the scores obtained in these quality-of-life domains.

## DISCUSSION

This study’s findings indicate a statistically significant correlation between the domains of the quality of life and sex only regarding the psychological domain; lower scores were found among women resulting from the workload experienced at the workplace and at home. Similar results were found in a cross-sectional and multicenter study conducted in Saudi Arabia with professionals working during the pandemic in ICUs, which showed that female workers obtained worse scores in the psychological domain than men due to the second shift^(13)^. On the other hand, a Brazilian study conducted in the State of Piauí found divergent data in which no statistically significant correlations were found between quality of life and demographic variables, such as sex^(17)^.

A study conducted in Malaysia found that income levels directly interfered with the quality of life scores, especially in the environment domain^(14)^. Another study conducted with health professionals at a COVID-19 testing center found that financial resources mainly impacted the social relationships domain^(18)^, which aligns with this study’s findings. The results found here indicate that income was directly and significantly associated with the environmental and social domains, as the salary levels offered by institutions in the city where the professionals were interviewed were below the general average wage. In addition, there is a study in which income negatively impacted the participants’ quality of life, i.e., professionals with less than four times the minimum wage obtained higher scores than those who reported an income above five times the minimum wage^(19)^.

Working hours are among the factors affecting quality of life. In this study, professionals working in more than one job work from 30 to more than 40 hours per week. Working in a second job meets the need for a higher income to ensure subsistence, consequently decreasing a worker’s overall quality of life. Thus, these findings corroborate those of Maqsood et al. (2021), in which workers working longer hours obtained lower scores, especially in general QoL^(13)^.

Furthermore, the concept of workload in this study concerns the total workload performed by an individual or collectively during a specific period^(20)^. In this sense, health professionals, especially ICU workers, faced a more significant workload during the pandemic. In addition to their regular workload, there was intense patient turnover and patients with multiple comorbidities, which taxed the workers in physical and psychological terms^(1)^.

A study conducted during the pandemic with ICU health professionals found an association between quality of life at work and workload. Although such an aspect is not in the scope of workload study, physical and psychological demands were affected by the number of environmental stressors^(21)^. This study shows that the greater the workload, the lower the scores obtained in the physical, psychological, and general domains concerning quality of life. More demands lead professionals to experience work overload, with repercussions beyond the work environment.

It is worth highlighting that work overload interferes with workers’ daily lives, especially in the workplace, leading to stress. When such a phenomenon surpasses the work environment’s barriers, interfering with life beyond work, it affects the quality of personal life^(22)^. A Vietnamese study aiming to assess the stress experienced by health professionals concluded that stress is associated with decreased quality of life scores in the physical, mental, social, and environmental domains^(4)^.

Moreover, Oswald (2012) found that the performance of health professionals in a health unit is significantly impacted by elements in the work environment, such as workspace and equipment^(23)^. This finding is corroborated by Sadatsafavi, Walewski, and Shepley (2015), who highlighted that structural and environmental resources (furniture, physical structure, and furnishings) contribute to professional health practices with satisfactory results^(24)^. This study also shows that the furniture assessment is related to the psychological domain, as the worse the workers assess the unit’s furniture, the lower the scores obtained in the psychological domain. For example, there was a need to adapt the environment’s infrastructure during the pandemic, and such changes failed to meet the work needs and demands.

Health professionals rely on technological equipment to support their practice and efficiently deliver care, such as infusion pumps, multiparameter monitors, and mechanical lung ventilators, among others, as provided in Resolution No. 7 from February 24, 2010^(25)^. Having proper functioning equipment available is a factor that impacts the lives of professionals. This study suggests that quality of life is also linked to equipment in the work environment, as the worse the workers assess the equipment in their units, the lower the scores obtained in the physical and psychological domains of quality of life; poorly functioning equipment hinders proper work processes. A similar study addressing primary health care professionals found a statistically significant correlation between technical equipment and work area, showing that technical equipment influences professional performance^(26)^.

### Study’s Limitations

As for this study’s limitations, conducting the study in other cities would expand the results’ analysis. Additionally, the sample did not allow exploring results according to each profession. Hence, addressing intensive care units in other cities would result in a more comprehensive study, allowing for an in-depth analysis of this topic by addressing other professions

### Contributions to the field of Nursing, Public Health, and Public Policy

This study’s findings contribute to advancing knowledge in the field of occupational health, enabling the identification of a knowledge gap. These results enable the implementation of interventions in work environments to reduce inequities that plague workers in terms of income, workload, furniture, and equipment. Furthermore, the results highlight the working conditions to which workers are exposed and, concomitantly, provide guidance on where public resources can be applied to improve the work environment.

## CONCLUSION

Health professionals, especially those working in intensive care units, suffered the consequences of the COVID-19 pandemic, facing several aspects harmful to QoL. Income, weekly working hours, workload, furniture, and equipment impacted QoL in at least one domain. Women were more predisposed to worse QoL scores compared to men, showing a need for future studies to focus on psychological issues and work overload due to the second shift.

Therefore, public health measures focused on occupational health are needed, especially among female professionals. Regarding salary, the nursing minimum wage was adjusted, which might improve income-related issues and, consequently, weekly working hours. As for workload, adequate staff dimensioning can also improve work routines. However, physical changes concerning the quality of furniture and equipment are also needed; inadequate equipment compromises care delivery, leading to workers’ dissatisfaction.
