# Supplementary material for: Sociodemographic and occupational influences on health professionals’ quality of life
Source: Rev Bras Enferm. 2024 Nov 22;77(4):e20240010. doi: 10.1590/0034-7167-2024-0010 (PMC11654231; doi:10.1590/0034-7167-2024-0010)
Supplement: Translation

# Translation: Influências sociodemográficas e ambientais de trabalho na qualidade de vida de profissionais de saúde

**DOI:** 10.1590/0034-7167-2024-0010pt

**Authors:** Alberto de Oliveira Redü, Daiani Modernel Xavier, Marcela Amaral Daoud, Pâmela Kath de Oliveira Nörnberg, Natália Sperli Geraldes Marin dos Santos Sasaki, Geovane Mendes Farias, Luciano Garcia Lourenção

**Affiliations:** I Universidade Federal do Rio Grande. Rio Grande, Rio Grande do Sul, Brasil; II Faculdade de Medicina de São José do Rio Preto. São José do Rio Preto, São Paulo, Brasil

**Keywords:** Qualidade de Vida, Pessoal da Saúde, Unidades de Terapia Intensiva, Condições de Trabalho, Trabalho

## Abstract

**Objetivo:**

Analisar as influências sociodemográficas e ambientais de trabalho na qualidade de vida de profissionais de saúde.

**Método:**

Tratou-se de um estudo descritivo-exploratório, transversal, analítico e quantitativo. Constituiu-se de 94 profissionais de saúde, sendo técnicos em enfermagem, enfermeiros e fisioterapeutas atuantes em Unidades de Terapia Intensiva, em um município do extremo sul do Brasil, no ano de 2023. Foi utilizado o teste de *t-student* e a Correlação de *Spearman*.

**Resultados:**

Houve correlação positiva significativa na variável sociodemográfica do sexo feminino com o domínio psicológico; renda associou-se positivamente com o domínio social e ambiente; horas de trabalho relacionou-se inversamente com a Qualidade de Vidas (QV) geral; volume de serviço interferiu negativamente no domínio físico, psicológico e QV geral; mobília influenciou, de maneira negativa, o domínio psicológico e equipamentos relacionou-se negativamente com domínio físico e psicológico.

**Conclusão:**

Verificou-se que as características do ambiente de trabalho interferem em diversos domínios da qualidade de vida.

## INTRODUÇÃO

No Brasil, os profissionais de saúde que atuam em Unidades de Terapia Intensiva (UTI) são constantemente submetidos a sobrecargas no trabalho, em razão da necessidade de desempenhar múltiplas funções^(1)^. O trabalho nesses ambientes é coordenado por tarefas complexas de atenção a pacientes críticos, os quais demandam cuidados específicos de alta complexidade, o que torna o modelo de cuidado individualizado, pautado nas necessidades e demandas do paciente^(2,3)^.

A partir do desenvolvimento econômico enfrentado nos últimos anos, os profissionais de saúde, além de desdobrarem-se para suprir as demandas de trabalho, ainda precisam realizar tarefas domésticas, destinando pouco tempo para atividades de lazer^(4)^. A sobrecarga de trabalho oriunda das condições laborais^(5)^, ambientes insalubres e que não disponham de equipamentos, materiais e infraestrutura^(6)^ necessária para atender as demandas tornam-se prejudiciais à saúde do trabalhador, o que contribui para a redução da qualidade de vida dos profissionais de saúde^(7)^.

A Qualidade de Vida (QV) é conceituada “como a percepção do indivíduo de sua posição na vida no contexto da cultura e sistema de valores, nos quais ele vive e em relação aos seus objetivos, expectativas, padrões e preocupações”^(8)^. Denota-se que o conceito se apresenta abrangente, permeando aspectos físicos, psicológicos, nível de independência, relações sociais e crenças pessoais^(9)^. Apesar de alguns estudos demonstrarem que a qualidade de vida dos profissionais de saúde apresenta, em geral, bons escores, ela foi altamente influenciada por questões como a COVID-19, em que houve uma descarga muito grande de agentes estressores, tornando significativa a relação entre o estresse e a qualidade de vida. Em virtude disso, percebe-se que a qualidade de vida foi alterada negativamente por fatores como a resiliência, fato esse que repercute também na qualidade da assistência prestada^(10,11)^.

Há evidências, na literatura mundial, acerca das questões do trabalho voltadas para a qualidade de vida. Países como África, Arábia Saudita, Malásia e Vietnã são detentores de publicações em que a qualidade de vida é colocada em xeque pelos fatores ambientais do local de trabalho, surtos de doenças como Ebola e COVID-19. A ameaça e o comprometimento da qualidade de vida provoca, de maneira intensa, dificuldades no enfrentamento das situações ambientais, como a grande demanda de pacientes e condições de infraestrutura adequadas, o que torna os profissionais predispostos a esgotamento e, a *posteriori*, Síndrome de Burnout (SB), estresse e baixa resiliência para lidar com as situações-problema do cotidiano^(4,12-14)^.

Diversos achados apontam para a qualidade de vida dos profissionais de saúde, além de variáveis relacionadas à SB, ao estresse, à resiliência e às estratégias para melhorar essas condições. Contudo, apesar disso, os estudos apresentam fragilidade por não demonstrar como as características socioambientais laborativas interferem no cotidiano dos profissionais fora do seu ambiente de trabalho. Para tal, parte-se do princípio de que as limitações do ambiente de trabalho interferem na maneira de desenvolver o trabalho, de atender os pacientes, de conseguir realizar com maior agilidade as demandas do setor de trabalho. Esses fatores repercutem na saúde física e mental dos profissionais, por trazerem o sentimento de impotência pelo fato de não conseguirem atender da maneira como gostariam, em razão da precariedade do ambiente.

Nesse contexto, a saúde constitui um dos pilares da sociedade atual, mas para que a saúde se desenvolva de maneira que atenda satisfatoriamente a população, os profissionais de saúde necessitam de condições adequadas de trabalho. Dessa forma, o ambiente de trabalho com condições desfavoráveis reflete na QV nos mais diversos domínios. Diante do exposto, o presente estudo teve a seguinte questão norteadora: Quais as influências sociodemográficas e ambientais de trabalho na qualidade de vida de profissionais de saúde?

## OBJETIVO

Analisar as influências sociodemográficas e ambientais de trabalho na qualidade de vida de profissionais de saúde.

## MÉTODOS

### Aspectos éticos

A pesquisa foi aprovada pelo Comitê de Ética em Pesquisa da Universidade Federal do Rio Grande (CEP-FURG), Certificado de Apresentação de Apreciação Ética (CAAE). Foi solicitada a anuência dos hospitais onde foi realizado o estudo, concedida por meio de formulário assinado pelos representantes dos setores responsáveis pelas pesquisas nas instituições. Os participantes que aceitaram participar do estudo assinaram o Termo de Consentimento Livre e Esclarecido (TCLE) em duas vias, ficando uma cópia com o participante e outra com o pesquisador responsável.

### Desenho do estudo e plano amostral

Realizou-se uma pesquisa descritivo-exploratório, transversal e correlacional de caráter quantitativo^(15)^. O estudo foi conduzido em dois hospitais do extremo sul do Brasil. O primeiro refere-se a um hospital universitário que apresenta uma UTI adulto que possui seis leitos, o segundo, um hospital filantrópico contendo três UTIs, uma UTI de atendimentos a convênios e particulares composta por 10 leitos, uma UTI geral contendo 10 leitos e uma Unidade Pós-Operatório destinada a serviços de cirurgia cardíaca e cuidados cardiológicos, com nove leitos.

Constituiu-se a população do estudo de 160 profissionais de saúde, aplicando-se, como critérios de inclusão, ser profissional técnico em enfermagem, enfermeiro ou fisioterapeuta da UTI. Foi adotado, como critério de exclusão, profissionais que estavam em férias ou licença saúde no período da coleta. Após a aplicação dos critérios, obtiveram-se 124 profissionais da saúde. Posteriormente, realizou-se o cálculo amostral por meio da ferramenta *StatCalc Epi Info* versão 7.2, com grau de confiança de 95% e margem de erro de 5%, resultando em uma amostra de 94 profissionais. Com o intuito de qualificar o presente estudo, o *Strengthening the Reporting of Observational Studies in Epidemiology* (STROBE) foi utilizado como instrumento norteador.

Diante do cenário pós-pandêmico em que foi realizado o estudo e levando em consideração que este é um recorte da dissertação intitulado “Dor cervical e qualidade de vida: repercussões em profissionais de saúde de unidade de terapia intensiva”, optou-se por incluir os profissionais de enfermagem e fisioterapia, uma vez que esses prestavam assistência direta e imediata. Nesse sentido, ambas as categorias profissionais experienciam rotinas semelhantes na questão de levantamento e deslocamento de paciente no leito e nas atividades rotineiras da unidade.

### Coleta dos dados

Os profissionais foram informados inicialmente sobre o estudo, por meio de uma mensagem enviada aos grupos de *WhatsApp* de cada unidade, além de serem convidados pessoalmente pelo pesquisador. A coleta de dados ocorreu por meio de um questionário autoaplicável de múltipla escolha, com tempo de aplicação em torno de 30 a 45 minutos, sendo realizado pelo pesquisador responsável nos meses de abril a agosto de 2023.

O questionário contemplou as variáveis sociodemográficas (função, sexo, idade, renda familiar, horas trabalhadas e tempo de atuação profissional) e variáveis características do ambiente de trabalho (volume de serviço, mobiliário, equipamentos e espaço físico), oriundo do Centro de Referência em Saúde do Trabalhador de Baía de Ilha Grande^(16)^. Além disso, como variável dependente, foi utilizada a versão abreviada do instrumento de qualidade de vida *Whoqol-Bref*, composto por 26 questões em escala *likert* de cinco pontos, dividida nos domínios físico, psicológico, meio ambiente, relações sociais e qualidade de vida geral^(9)^.

Para verificar se os questionários apresentavam valor semântico e estrutural adequados ao que era proposto no estudo, foi aplicado um teste piloto com profissionais de um turno de trabalho em uma das unidades do estudo. Posteriormente, não havendo necessidade de reformulação, incorporaram-se esses participantes à amostra.

### Análise dos dados

Os dados foram digitados e tabulados no *software* estatístico *Statistical Package for the Social Sciences* (SPSS), versão 28.0. As variáveis quantitativas foram descritas por média e desvio-padrão ou mediana e amplitude interquartílica, dependendo da distribuição dos dados.

Na análise do *Whoqol-bref,* realizou-se o critério da soma das questões relativas ao domínio analisado, após dividiu-se pela quantidade de questões do domínio. Vale destacar que, para o cálculo, foi utilizada a recodificação das questões 3, 4 e 26^(9)^.

Para realizar a testagem da normalidade dos dados, foi utilizado o teste de *Kolmogorov-Smirnov*. Em seguida, as variáveis foram descritas por frequências absolutas e relativas e, para comparar as médias, o teste *t-student* foi utilizado. Como as variáveis apresentavam caráter ordinal, foi utilizado o teste de Correlação de *Spearman* com o intuito de avaliar associação entre elas. Adotou-se como nível de significância 5% (p<0,05).

## RESULTADOS

Participaram da pesquisa 94 profissionais da saúde. A Tabela 1 expressa a caracterização amostral dos profissionais que, em sua maioria, 88,3% eram mulheres, 34,1% apresentavam uma faixa etária de 30 a 39 anos de idade e 31,9% tinham uma renda familiar de 1 a 2 salários mínimos. Quanto às horas de trabalho semanal, 67% realizavam de 36 a 40 horas semanais, 38,3% atuavam de 1 a 5 anos na profissão e 63,8% eram da categoria de técnicos de enfermagem.

**Tabela 1:** Caracterização amostral dos profissionais de saúde atuantes nas unidades de terapia intensiva. Rio Grande, Rio Grande do Sul, Brasil, 2023 (n=94)

Variáveis	n	%
Sexo		
Masculino	11	11,7
Feminino	83	88,3
Faixa etária		
20 a 29 anos	25	26,6
30 a 39 anos	32	34,1
40 a 50 anos	31	33,0
Superior a 50 anos	6	6,4
Renda familiar		
Entre 1 e 2 s.m.	30	31,9
Entre 2 e 3 s.m.	22	23,4
Entre 3 e 5 s.m.	23	24,5
Entre 5 e 20 s.m.	20	20,2
Horas de trabalho semanal		
20 a 30 horas	18	19,1
36 a 40 horas	57	67,0
Maior que 40 horas	13	13,8
Tempo de atuação profissional		
0 a 12 meses	9	9,6
1 a 5 anos	36	38,3
6 a 9 anos	15	16,0
Maior que 10 anos	34	36,2
Categoria profissional		
Técnico de Enfermagem	60	63,8
Enfermeiro	21	22,3
Fisioterapeuta	13	13,8

*Nota: s.m.: Salário Mínimo R$1.320,00 ≈ USD 270.49 (1 USD = R$4,88)*

A Tabela 2 apresenta as variáveis referentes ao ambiente de trabalho dos profissionais as quais foram quantificadas por qualificadores. Dessa forma, 52,1% dos profissionais avaliaram que existe uma sobrecarga em relação ao volume de serviço, 48,9% avaliaram a mobília como regular e a avaliação dos equipamentos do ambiente de trabalho variou entre bom e regular, com 44,7 cada um.

**Tabela 2:** Caracterização do ambiente de trabalho dos profissionais de saúde atuantes nas unidades de terapia intensiva. Rio Grande, Rio Grande do Sul, Brasil, 2023 (n=94)

Variáveis	n	%
Como você avalia o volume de serviço?		
Leve	1	1,1
Moderado	35	37,2
Existe uma sobrecarga	49	52,1
Exaustivo	9	9,6
Como você avalia a mobília no seu setor de serviço?		
Excelente	2	2,1
Boa	34	36,2
Regular	46	48,9
Ruim	11	11,7
Muito ruim	1	1,1
Como você avalia a qualidade dos equipamentos do seu setor de trabalho?		
Excelente	4	4,3
Boa	42	44,7
Regular	42	44,7
Ruim	6	6,4

Para a análise dos domínios do *Whoqol-Bref,* ocorreu uma variação média entre 58,9 e 68,1, conforme a Tabela 3. O domínio que apresentou menor escore foi o meio ambiente (58,9), composto pelas facetas: Segurança física e proteção; Ambiente no lar; Recursos financeiros; Cuidados de saúde e sociais; Disponibilidade e qualidade; Oportunidades de adquirir novas informações e habilidade; Participação em/e oportunidades de recreação/lazer; Ambiente físico, (poluição/ruído/trânsito/clima) e transporte. O domínio que apresentou maior escore foi o social (68,1), originado pelas seguintes facetas: Relações pessoais; Suporte (Apoio) social e atividade sexual.

**Tabela 3:** Escores médios dos domínios do *Whoqol-Bref.* Rio Grande, Rio Grande do Sul, Brasil, 2023 (n=94)

Domínios	Média ± DP	Mínimo - Máximo
Físico	64,6 ± 13,5	35,7 - 100
Psicológico	65,7 ± 15,1	25,0 - 95,8
Relações Sociais	68,1 ± 18,5	25,0 - 100
Meio Ambiente	58,9 ± 13,5	28,1 - 90,6
Geral	60,1 ± 19,3	12,5 - 100

*DP: Desvio-padrão.*

Quando comparados os escores de qualidade de vida entre os sexos dos participantes, houve diferença significativa apenas no domínio psicológico (p=0,017) no qual se verificou menores escores nas mulheres. As demais diferenças não foram significativas entre os sexos (p>0,30), conforme pode ser visualizado na Figura 1.

**Figura 1:** Avaliação dos escores de qualidade de vida conforme sexo dos participantes. Rio Grande, Rio Grande do Sul, Brasil, 2023 (n = 94)

A Tabela 4 apresenta os resultados das associações entre as variáveis sociodemográficas e relacionadas ao trabalho com os domínios do *Whoqol-bref*. Houve associação direta estatisticamente significativa entre a renda e os domínios social e ambiente do *Whoqol-Bref*, ou seja, quanto maior a renda, maiores os escores nesses domínios. Horas de trabalho associou-se inversamente com os escores gerais de qualidade de vida, ou seja, quanto maior o tempo de trabalho, menores os escores gerais do *Whoqol-Bref.*

**Tabela 4:** Associações entre os escores de qualidade de vida com as variáveis sociodemográficas e relacionadas ao trabalho. Rio Grande, Rio Grande do Sul, Brasil, 2023 (n=94)

Variáveis	Domínios do *WHOQOL-BREF*				
	Físico	Psicológico	Social	Ambiente	Geral
Idade	-0,09	0,14	-0,07	-0,09	-0,06
	(p=0,372)	(p=0,171)	(p=0,516)	(p=0,380)	(p=0,554)
Renda familiar	0,08	0,19	0,26	0,20	0,11
	(p=0,437)	(p=0,060)	(p=0,011)	(p=0,049)	(p=0,282)
Horas de trabalho	-0,05	-0,17	-0,12	-0,10	-0,27
	(p=0,655)	(p=0,112)	(p=0,253)	(p=0,349)	(p=0,009)
Tempo de atuação	-0,06	0,09	0,04	-0,01	-0,02
	(p=0,559)	(p=0,400)	(p=0,696)	(p=0,900)	(p=0,873)
Volume de serviço^*^	-0,25	-0,20	-0,18	-0,17	-0,31
	(p=0,015)	(p=0,049)	(p=0,081)	(p=0,093)	(p=0,002)
Mobília no seu setor de serviço^*^	-0,18	-0,26	-0,18	-0,09	-0,10
	(p=0,092)	(p=0,012)	(p=0,075)	(p=0,399)	(p=0,337)
Equipamentos do seu setor de trabalho^*^	-0,22	-0,28	-0,12	-0,14	-0,17
	(p=0,030)	(p=0,006)	(p=0,251)	(p=0,191)	(p=0,110)
Espaço físico do seu ambiente de trabalho^*^	0,04	0,09	-0,03	-0,04	-0,01
	(p=0,677)	(p=0,399)	(p=0,742)	(p=0,735)	(p=0,916)

Nota: valores dos coeficientes de correlação de Spearman inseridos na tabela;

* quanto maior, pior a avaliação.

Também houve associação inversa significativa entre a avaliação do volume de serviço e os domínios físico, psicológico e geral de qualidade de vida, indicando que quanto maior o volume do serviço, menores esses escores de qualidade de vida. Além disso, houve associação inversa significativa entre a avaliação da mobília no setor de serviço e o domínio psicológico de qualidade de vida, demostrando que quanto pior a avaliação da mobília (valores mais altos), menores os escores neste domínio da qualidade de vida (valores mais baixos). Por fim, houve associação inversa significativa entre a avaliação dos equipamentos no setor de serviço e os domínios físico e psicológico de qualidade de vida, indicando que quanto pior a avaliação dos equipamentos, menores os escores nestes domínios da qualidade de vida.

## DISCUSSÃO

Nos achados do presente estudo, após a análise de correlação dos domínios do questionário da qualidade de vida com a variável sexo, constatou-se que houve diferença estatística significativa apenas no domínio psicológico, no qual se verificou menores escores nas mulheres ocasionados pela carga de trabalho acarretada pelo trabalho doméstico somado ao ocupacional. Resultados semelhantes foram encontrados em estudo transversal e multicêntrico conduzido na Arábia Saudita com profissionais que atuaram durante a pandemia, em unidades de terapia intensiva, o qual demonstrou que as profissionais do sexo feminino apresentavam piores escores do domínio psicológico quando comparados com os dos homens, em função da dupla jornada de trabalho^(13)^. Por outro lado, um estudo brasileiro conduzido no estado do Piauí encontrou dados divergentes, em que as correlações de qualidade de vida com variáveis demográficas, como sexo, não apresentaram significância estatística^(17)^.

No que se refere à renda, estudo conduzido na Malásia encontrou resultados mostrando que o nível de renda interferiu diretamente nos escores de qualidade de vida, em especial, no domínio ambiente^(14)^. Em outra pesquisa conduzida com profissionais de saúde em um centro de testagem para COVID-19, verificou-se que, prioritariamente, os recursos financeiros impactaram o domínio de relações sociais^(18)^. Esses achados vão ao encontro dos dados do presente estudo, em que a renda apresentou associação direta e significativa com os domínios ambiente e social, visto que as condições salariais ofertadas pelas instituições da cidade na qual os profissionais foram investigados estão abaixo da média geral salarial das categoriais profissionais. Contudo, há um estudo em que se constatou que a renda interferiu de maneira negativa na qualidade de vida dos participantes, uma vez que os profissionais com renda inferior a quatro salários mínimos apresentavam maiores escores em relação aos que referiram renda superior a cinco salários mínimos^(19)^.

Entre os fatores que interferem na qualidade de vida, há ainda a quantidade de horas trabalhadas. Neste estudo, profissionais que atuavam em mais de um emprego, apresentando jornada semanal alterada variando de 30 a mais de 40 horas semanais de trabalho, diante da necessidade de melhor renda para manutenção da subsistência, consequentemente, apresentaram menor qualidade de vida geral. Assim, esses achados corroboram os de Maqsood et al. (2021), em que os trabalhadores que tinham mais horas de trabalho apresentavam escores menores, sobretudo da QV geral^(13)^.

Ademais, no presente estudo, o conceito de volume de trabalho assemelha-se ao de carga de trabalho, em que o trabalho total é desenvolvido de maneira individual e/ou coletiva, durante um período específico^(20)^. Nesse sentido, nota-se que os profissionais de saúde, em especial os de UTI, apresentaram uma sobrecarga de trabalho maior durante a pandemia, uma vez que experimentaram, além da carga de trabalho com intensa rotatividade de pacientes, pacientes com múltiplas comorbidades, entre outros fatores que afetaram não somente o físico mas também o psicológico dos profissionais^(1)^.

Em um estudo realizado no período pandêmico com profissionais de saúde de UTI, houve associação entre a qualidade de vida no trabalho e a carga de trabalho. Apesar de não ser o escopo de estudo de carga de trabalho, o volume de trabalho, no que se refere às demandas físicas e psicológicas, apresentou-se alterado em razão da quantidade de estressores ambientais^(21)^. O presente estudo, demonstra que quanto maior o volume de serviço, menores os escores de qualidade de vida no domínio físico, psicológico e geral. Esse fato demonstra que as demandas de trabalho aumentadas geram sobrecarga ao profissional, ocasionando repercussões fora do ambiente laboral.

Vale destacar que a ocorrência da carga de trabalho aumentada interfere no cotidiano dos profissionais de saúde, em especial, no seu ambiente laboral. Isso contribui para o desenvolvimento de estresse, porém, quando esse fenômeno ultrapassa as barreiras do ambiente de trabalho e passa a interferir na vida além dele, promove repercussões na qualidade de vida pessoal^(22)^. Em um estudo vietnamita cujo objetivo foi avaliar os profissionais da saúde acerca do estresse concluiu que este está associado à diminuição dos escores de qualidade de vida nos domínios físico, mental, social e ambiental^(4)^.

Destaca-se, ainda, no estudo de Oswald (2012), que o desempenho dos profissionais de saúde em uma unidade desse tipo é significativamente impactado por elementos do ambiente de trabalho, como espaço e equipamentos^(23)^. Corrobora esse achado Sadatsafavi, Walewski, Shepley (2015), os quais ressaltaram que os recursos estruturais e ambientais (móveis, construção e acabamentos) contribuem para práticas profissionais em saúde, com resultados satisfatórios^(24)^. Ao encontro disso, nos achados do presente estudo, a avaliação da mobília do setor de trabalho relaciona-se com o domínio psicológico da qualidade de vida, uma vez que, quanto pior a avaliação da mobília, menor os escores nesse domínio, visto que, durante o período pandêmico, houve a necessidade de adequações de infraestrutura do ambiente que não atendia as necessidades das demandas de trabalho.

Para desenvolver seu trabalho de maneira eficiente, os profissionais de saúde contam com uma série de equipamentos tecnológicos que oferecem suporte ao seu atendimento, como bombas de infusão, monitores multiparamétricos, ventiladores pulmonares mecânicos, entre outros equipamentos dispostos na Resolução nº 7 de 24 de fevereiro de 2010^(25)^. O funcionamento adequado dos equipamentos, além da disponibilidade deles pelas instituições de saúde, demonstra ser um fator que impacta na vida dos profissionais. Este estudo sugere que a qualidade de vida está atrelada também aos equipamentos do ambiente de trabalho, uma vez que quanto pior a avaliação dos equipamentos, menor são os escores dos domínios físico e psicológico da qualidade de vida, em virtude de essa precarização dificultar o processo de trabalho. Em um estudo semelhante com profissionais de saúde, porém no contexto da atenção primária, houve uma correlação estatisticamente significativa entre os equipamentos técnicos com a atuação e área de trabalho, o que demonstrou que o desempenho profissional foi influenciado pelos equipamentos técnicos^(26)^.

### Limitações do estudo

Como limitações do estudo, aponta-se que a realização do estudo em outros municípios ampliaria a análise dos resultados. A amostra não permitiu a exploração dos resultados segundo a categoria profissional. Sugere-se que a realização em mais unidades de terapia intensiva de outros municípios tornaria o estudo mais abrangente, permitindo realizar um aprofundamento maior acerca dessa temática, com o enfoque nas demais categorias profissionais.

### Contribuições para a área da Enfermagem, saúde ou política pública

Diante disso, os achados do presente estudo contribuem para o avanço do conhecimento na área da saúde do trabalhador e, dessa forma, permitiram encontrar uma lacuna do conhecimento. Esses resultados permitem futuramente a possibilidade de realizar intervenções nos ambientes laborativos, de modo que sejam reduzidas as iniquidades que assolam os trabalhadores quanto às questões voltadas à renda, ao volume de serviço, ao mobiliário e aos equipamentos. Além disso, constituem uma maneira de evidenciar as condições de trabalho às quais os trabalhadores são expostos e, concomitantemente, o direcionamento de recursos públicos para o melhoramento do ambiente de trabalho.

## CONCLUSÕES

Os profissionais de saúde, em especial os atuantes nas unidades de terapia intensiva, sofreram as consequências da pandemia de COVID-19, enfrentando diversos agentes prejudiciais à QV. Diante disso, fatores como renda, horas de trabalho semanal, volume de serviço, mobília e equipamentos são destaques que repercutiram na QV, em pelo menos um domínio. As mulheres apresentaram maior predisposição para piores escores de QV quando comparadas aos homens. Demonstra-se, a partir disso, a necessidade de novos estudos que promovam um enfoque para as questões psicológicas e a sobrecarga em razão da dupla jornada de trabalho.

Nesse sentido, tornam-se necessárias medidas de saúde pública que direcionem a atenção para a saúde do trabalhador, especialmente, para as profissionais do sexo feminino. Quanto à questão salarial, o piso salarial da enfermagem promoveu um reajuste para os profissionais, o que pode contribuir para melhorar as questões voltadas para a renda e, consequentemente, as horas de trabalho semanais. No que se refere ao volume de serviço, sugere-se que um dimensionamento de profissionais adequado possa melhorar as rotinas da unidade. No entanto, são necessárias também mudanças físicas em questões, como o mobiliário e o equipamento, uma vez que, se esses equipamentos apresentam inadequações, comprometem a forma de assistência prestada e, concomitantemente, também promovem insatisfação no trabalhador que desempenha a função.
